# The inhibition of malignant melanoma cell invasion of bone by the TLR7 agonist R848 is dependent upon pro-inflammatory cytokines produced by bone marrow macrophages

**DOI:** 10.18632/oncotarget.25711

**Published:** 2018-07-06

**Authors:** Yoko Manome, Dai Suzuki, Ayako Mochizuki, Emi Saito, Kiyohito Sasa, Kentaro Yoshimura, Tomio Inoue, Masamichi Takami, Katsunori Inagaki, Takahiro Funatsu, Ryutaro Kamijo

**Affiliations:** ^1^ Departments of Biochemistry School of Dentistry, Showa University, Shinagawa, Tokyo, Japan; ^2^ Department of Special Needs Dentistry, Division of Dentistry for Persons with Disabilities, Showa University Dental Hospital, Shinagawa, Tokyo, Japan; ^3^ Departments of Oral Physiology School of Dentistry, Showa University, Shinagawa, Tokyo, Japan; ^4^ Department of Orthopedic Surgery School of Medicine, Showa University, Shinagawa, Tokyo, Japan; ^5^ Departments of Pharmacology School of Dentistry, Showa University, Shinagawa, Tokyo, Japan

**Keywords:** bone invasion, bone marrow macrophage, cytokines, malignant melanoma, R848

## Abstract

Distant metastasis remarkably worsens the prognoses of malignant melanoma patients. Toll-like receptors (TLRs) recognize molecules derived from many types of pathogens and activate the innate intravital immune system. In this study, we examined the effects of R848, a TLR7 ligand, on bone invasion by malignant melanoma cells. Mice underwent transplantation with cells of a malignant melanoma cell line B16F10, and were also administered R848 every three days. Hindlimbs were obtained 13 days after transplantation and invasion of bone marrow by B16F10 cells was evaluated. ELISA was used to determine the concentrations of cytokines in mouse serum and in the culture medium from bone marrow macrophages (BMMs) in the presence or absence of R848. In addition, MTS assays were used to examine the effects of media from BMM cultures on the proliferation of B16F10 cells. The rate of infiltration by B16F10 cells and the area of invasion were significantly reduced with R848 administration. Furthermore, serum levels of IL-6, IL-12, and IFN-γ were significantly increased in mice administered R848, with the same trend observed in the culture medium of BMMs treated with R848. In addition, B16F10 cell proliferation was suppressed by the addition of medium from cultured BMMs treated with R848. Neutralization by antibodies against IL-6, IL-12, and IFN-γ abrogated the suppression of proliferation of B16F10 cells by culture medium from BMMs treated with R848. Our results suggest that R848 drives the production of IL-6, IL-12, and IFN-γ in BMMs, which reduces proliferation and bone invasion by B16F10 cells.

## INTRODUCTION

Malignant melanoma, one of the most dangerous types of skin cancer, develops from melanocytes, though it rarely occurs in the mouth, intestines, or eyes. In 2012, the number of individuals affected by melanoma worldwide was 232,000 and there were 55,000 associated deaths [1]. Typically, the treatment of a malignant melanoma includes its surgical removal. Most patients are cured if no metastasis has occurred, with the 5-year survival rates of Stage 0 and I cases being greater than 90% [2]. In contrast, survival of patients with Stage III (regional metastasis) is only approximately 50% and of those with Stage IV (distant metastasis) less than 10%, as cancer can metastasize to the lungs, brain, liver, and/or bone. The median survival of patients affected by bone metastasis ranges from 4 to 6 months [3], thus metastasis of a malignant melanoma to bone is a late event in the evolution of this disease. In those cases, pain is the most common presenting symptom and palliation is often the treatment goal. However, survival of patients with spreading metastatic malignant melanoma may be improved by treatments such as chemotherapy including with dimethyl triazeno imidazole carboxamide (DTIC) and dacarbazine; immunotherapy including with interleukin (IL)-2; targeted therapy including with BRAF inhibitors such as vemurafenib and dabrafenib; and radiation therapy. Furthermore, postoperative adjunctive biological therapy with interferon (IFN)-α has been shown to have a sustained effect on recurrence-free survival.

Recently, the most effective treatment for patients with advanced malignant melanoma is reported to be immune checkpoint therapy using the anti-PD-1 (programmed cell death 1) antibodies nivolumab and pembrolizumab, and/or the anti-CTLA-4 (cytotoxic T-lymphocyte-associated antigen 4) antibody ipilimumab, as the response rate of a combination of these antibodies has been reported to be 40-50% [4]. However, administration of the anti-CTLA-4 antibody often results in immunological side-effects and the cost is quite expensive. Therefore, a novel effective therapy for metastatic malignant melanoma is necessary to provide another choice of treatment for patients.

Toll-like receptors (TLRs) recognize pathogen-associated molecular patterns and play important roles in the innate immune system. In response to TLR ligands, immune cells produce pro-inflammatory cytokines to activate the immune system. Among TLR family members, TLR7 recognizes viral single-stranded RNAs (ssRNAs) and imidazoquinoline compounds, including imiquimod (R837) and resiquimod (R848) [5]. Clinically, TLR-targeting drugs including imidazoquinoline compounds, are speculated to show anti-tumor effects by activating the immune system [6]. Another imidazoquinoline compound, 852A, induces IFN-α from plasmacytoid dendritic cells (pDCs), and it was previously shown that 852A-induced IFN-α inhibits proliferation of the malignant melanoma cell line B16F10 and renal cell adenocarcinoma cells, as well as lung colonization by B16F10 cells in mice [7]. Also, both imiquimod and IFN-α-stimulated pDCs were reported to lyse the malignant melanoma cell lines SKMel2 and WM793 in a tumor necrosis factor-related apoptosis inducing ligand (TRAIL)-dependent fashion [8].

It is predicted that imidazoquinoline compounds might produce greater anti-tumor effects via activation of the immune system, especially in bones which have many immune cells [9]. However, their effect on bone invasion by melanoma cells has not been elucidated. In our study, we investigated whether the imidazoquinoline compound R848 could be utilized as a therapeutic option for malignant melanoma invasion of bone tissue using a mouse metastasis model and B16F10 cells. Our results suggest that R848 provides effective protection against the invasion of bine tissue by malignant melanoma cells via the induction of pro-inflammatory cytokines from bone marrow macrophages.

## RESULTS

**Figure 1:**
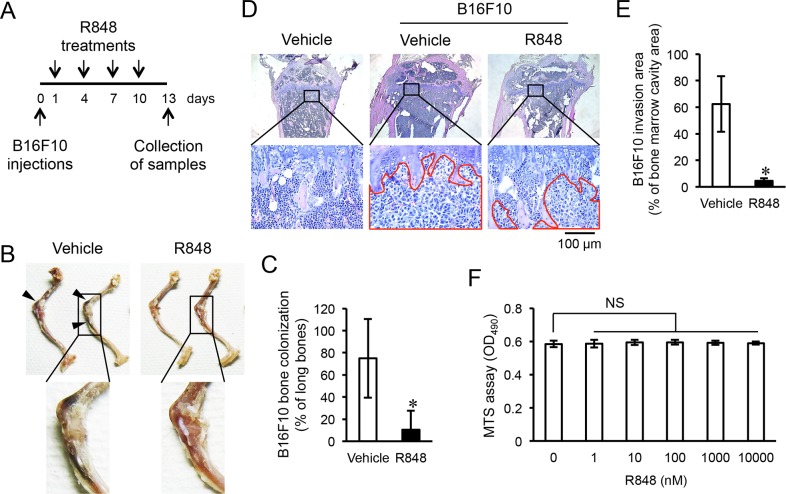
R848 inhibits invasion of epiphyseal bone by B16F10 cells **(A)** The protocol used for transplantation of malignant melanoma cell line B16F10 and R848 treatments. **(B)** Representative images showing invasion in epiphyseal bone in a hindlimb initiated by intracardiac injection of B16F10 cells. Arrowheads indicate the accumulation of those cells in epiphyseal bone. **(C)** Scoring for the invasion of epiphyseal bone in hindlimbs following treatment with the vehicle (DMSO) or R848 (500 μg/mL) (n=7 in each group). **(D)** Representative histology of tibia mesial epiphyses stained with HE. The red enclosed area shows B16F10 cell invasion. **(E)** Comparisons of areas of invasion in bone marrow cavities following treatment with the vehicle or R848. **(F)** MTS assay of B16F10 cells cultured with the indicated doses of R848 for one day. The data are representative of more than three independent experiments. ^*^p <0.01; NS, not significant.

To determine whether R848 results in toxicity, we measured body weights following the intraperitoneal injection of R848 (500 μg) every three days, though no body-weight effect was found (Supplementary Figure 1A). Next, we investigated whether the administration of R848 inhibited cancer invasion into bone. Following intracardiac injections of the malignant melanoma cell line B16F10, which created a murine metastasis model, mice were treated with intraperitoneal injections of R848 every three days (Figure 1A). We then dissected the hindlimbs at 13 days after B16F10 cell transplantation and evaluated cancer cell colonization and invasion levels in epiphyseal bone. Accumulations of blackened B16F10 cells were observed in the epiphyses of the femora and tibiae in mice treated with the vehicle (Figure 1B), while these accumulations were markedly reduced in mice treated with R848 (Figure 1B, 1C). Additionally, histological analyses of tibia medial epiphyses revealed the presence of fusiform-shaped B16F10 cells throughout the bone marrow cavity of mice that received those cells and the vehicle. Bone marrow cells, which were granular, filled the bone marrow cavity of mice that received vehicle without B16F10 cells (Figure 1D). Conversely, the area of invasion of B16F10 cells was significantly suppressed by treatment with R848 (Figure 1D, 1E). Moreover, mice transplanted with B16F10 cells showed reduced body weights at 9 and 12 days after intracardiac injections (Supplementary Figure 1B), but treatment with R848 did not results in recovery of lost body weight (Supplementary Figure 1C). In those samples, we investigated the bone morphology and measured the trabecular bone volume in tibiae from the proximal sides using micro-computed tomography (μCT). There were no significant differences in regard to bone volume per tissue volume (BV/TV) between the absence (−) and presence (+) of B16F10 cells, or between treatments with the vehicle and R848 (Supplementary Figure 2). Subsequently, to investigate whether R848 directly affects B16F10 cells, we treated cells with various concentrations of R848 and evaluated cellular proliferation using an MTS assay. However, even a high concentration of R848 had no effect on B16F10 cell proliferation (Figure 1F). These findings suggest that R848 indirectly reduced B16F10 cell invasion of epiphyseal bone in the present *in vivo* setting.

**Figure 2:**
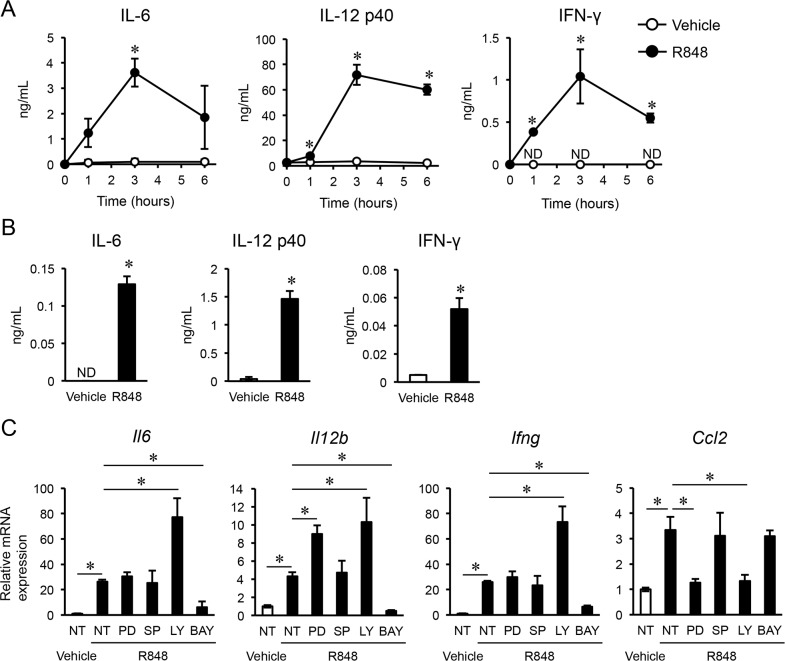
R848 induces pro-inflammatory cytokines **(A-B)** Concentrations of IL-6, IL-12 p40, and IFN-γ determined by ELISA in serum extracted from mice after intraperitoneal injections of the vehicle or R848 (500 μg/mL) (n=3 in each group) (A), and in media of bone marrow macrophage cultures treated with the vehicle or R848 (100 nM) (B). **(C)** Quantitative RT-PCR analysis of *Il6*, *Il12b*, *Ifng*, and *Ccl2* mRNA expressions in bone marrow macrophages not treated (NT) or treated with inhibitors (PD, PD98059; SP, SP600125; LY, LY294002; BAY, BAY11-7082) one hour before culturing with vehicle or R848 (100 nM). The data are representative of more than two independent experiments. ^*^p <0.01; ND, not detected.

**Figure 3:**
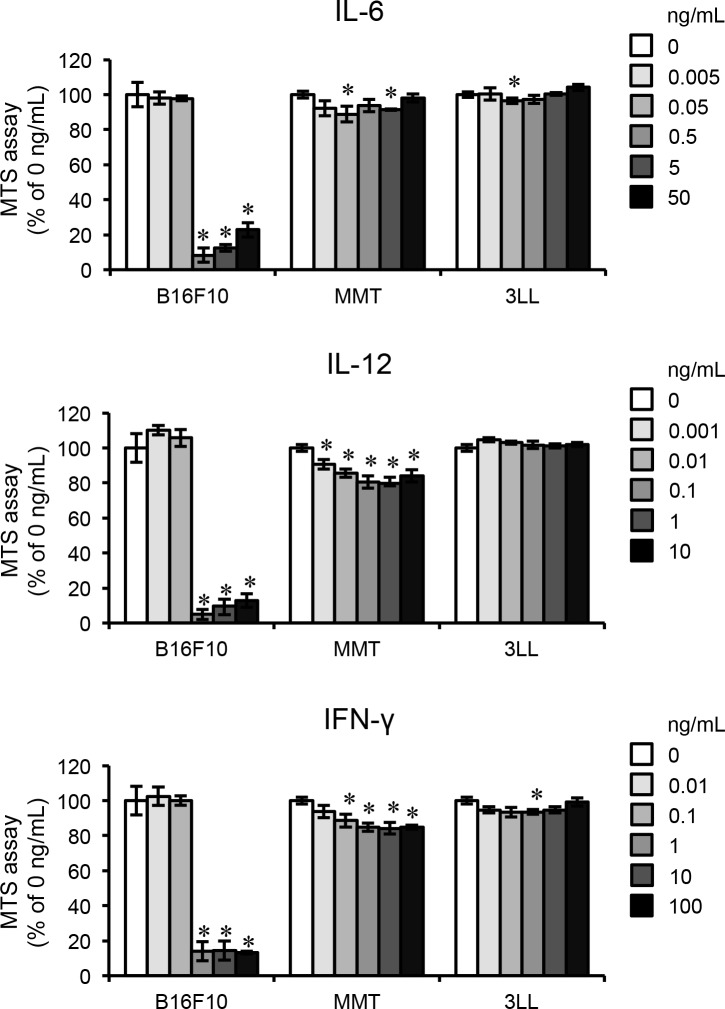
Pro-inflammatory cytokines inhibit proliferation of B16F10 MTS assays of cultures of B16F10 cells, the mammary tumor cell line MMT060562 (MMT), and the lung carcinoma cell line Ex-3LL (3LL) treated with the indicated doses of IL-6, IL-12, and IFN-γ for one day. The data are representative of more than three independent experiments. ^*^p <0.01.

Previous reports have noted that TLR agonists including R848 have effects on immune cells, such as macrophages and dendritic cells, stimulating them to induce pro-inflammatory cytokines. Various types of induced pro-inflammatory cytokines, such as IL-12 and IFN-γ, have anti-cancer functions [10, 11]. Therefore, we measured the concentrations of these cytokines in serum obtained from mice that received intraperitoneal injections of R848 using ELISA. We found that IL-6, IL-12 p40, and IFN-γ were significantly increased in the R848-treated group as compared with the vehicle group, with the peak at three hours after injection (Figure 2A). To determine whether immune cells were the source of these cytokines, we then evaluated the concentrations of IL-6, IL-12 p40, and IFN-γ in the supernatant of BMMs cultured with R848 (100 nM). Those results showed that their levels were significantly increased by R848 as compared with the vehicle treatment (Figure 2B). To investigate signal pathways that lead to increased levels of the previously mentioned cytokines and C-C motif chemokine 2 (CCL2), also known as monocyte chemoattractant protein 1 (MCP1), using kinase inhibitors. Therefore, we found that BAY 11-7082, an NF-κB inhibitor, suppressed the up-regulation of *Il6*, *Il12*, and *Ifng* expression by R848 (Figure 2C). Furthermore, PD98059, an ERK inhibitor, and LY294002, a PI3 kinase inhibitor, suppressed the up-regulation of *Ccl2* by R848. These results suggest that R848 increases the expressions of *Il6*, *Il12*, and *Ifng* through the NF-κB pathway, and *Ccl2* expression through the ERK and PI3 kinase pathways. To examine whether these cytokines have effects on cancer cells, we examined the proliferation of typical types of cancer cells cultured with various concentrations of recombinant IL-6, IL-12, and IFN-γ using an MTS assay. We found that a concentration of greater than 0.5 ng/mL of IL-6, 0.1 ng/mL of IL-12, and 1 ng/mL of IFN-γ dramatically suppressed B16F10 cell proliferation, whereas those cytokines had minimal effects on MMT, mammary tumor cells, and 3LL, which are lung carcinoma cells (Figure 3).

**Figure 4:**
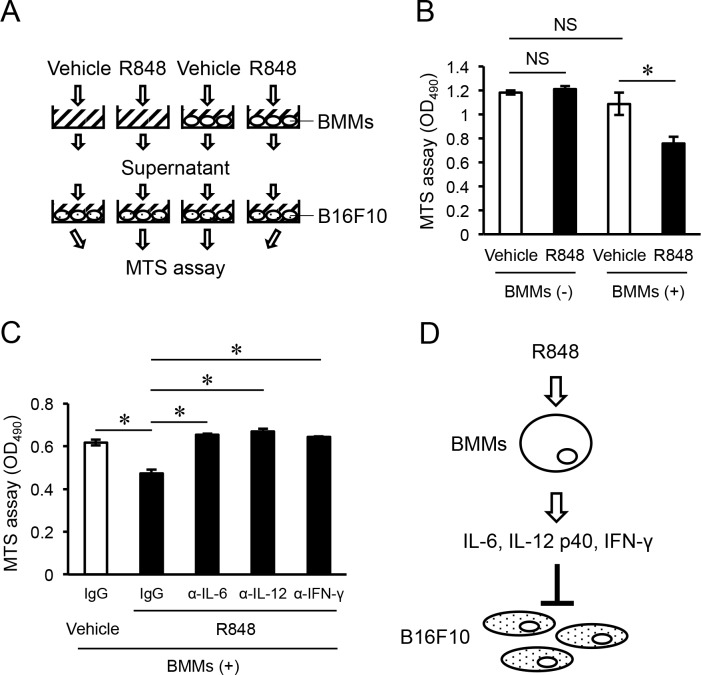
Pro-inflammatory cytokines induced from R848-treated BMMs inhibit proliferation of B16F10 cells **(A)** Schematic representation of the protocol for the MTS assay of B16F10 cell cultures. **(B)** MTS assay of B16F10 cultures in medium supernatant with (+) or without (−) bone marrow macrophages (BMMs), and treatment with the vehicle or R848 (100 nM) (right graph). **(C)** MTS assay of B16F10 cultures in medium supernatant from BMMs cultured with the vehicle or R848 (100 nM), and neutralizing antibodies to IL-6, IL-12, and IFN-γ. **(D)** Model for the inhibition of B16F10 cell proliferation by IL-6, IL-12, and IFN-γ induced from BMMs by treatment with R848. The data are representative of more than three independent experiments. ^*^p <0.01; NS, not significant.

To determine whether these cytokines induced from BMMs by R848 had effects on the proliferation of B16F10, we used an MTS assay to examine B16F10 cells cultured with supernatant obtained from cultures without or with BMMs and treated with the vehicle or R848 for one day (Figure 4A). Supernatant from cultured BMMs with added R848 inhibited the proliferation of B16F10 cells as compared with BMM culture supernatant with only added vehicle, while there were no significant differences between supernatant from cultures with or without BMMs when the vehicle was added (Figure 4B). Finally, to determine whether those cytokines in supernatant had an inhibitory effect on the proliferation of B16F10 cells, we added the neutralizing antibodies to IL-6, IL-12, and IFN-γ to the cultures. This treatment abrogated the inhibition of cell proliferation induced by supernatant from BMMs cultured with R848 (Figure 4C). Our findings suggest that BMMs secrete IL-6, IL-12 p40, and IFN-γ in response to R848 treatment, and those cytokines inhibit the proliferation of B16F10 cells.

## DISCUSSION

In tour study, R848 induced the production of IL-6, IL-12, and IFN-γ from BMMs *in vitro* (Figure 2B). In addition, recombinant cytokines and supernatants from cultured BMMs stimulated with R848 suppressed proliferation of the malignant melanoma cell line B16F10 (Figures 3, 4B). Suppression of B16F10 proliferation by BMM supernatant stimulated by R848 was negated by neutralizing antibodies to IL-6, IL-12, and IFN-γ (Figure 4C). Moreover, R848 did not directly suppress B16F10 proliferation *in vitro* (Figure 1F). Together, these results suggest that R848 indirectly inhibits B16F10 proliferation via BMM-produced cytokines (Figure 4D). Although the mechanism by which R848 inhibits bone invasion by B16F10 cells *in vivo* has not been fully elucidated, it is highly possible that the *in vivo* mechanism is similar to the *in vitro* model, because R848-injected mice showed increased serum levels of IL-6, IL-12, and IFN-γ (Figure 2A).

Our study is the first to report that imidazoquinoline compounds are effective for treatment of bone invasion by malignant melanoma cells. Bone resorption inhibitors, including bisphosphonates, are frequently used to treat bone invasion after cancer metastasis to bone tissue. R848 has been shown to inhibit osteoclast differentiation *in vitro* [12] and the same effect is expected *in vivo*. However, our results showed that bone volume in R848-treated mice was unchanged (Supplementary Figure 2), which suggests that the numbers and/or activities of osteoclasts in bone were not altered. These observations indicate that the mechanism of R848 suppression of malignant melanoma cell bone invasion is unlikely to be related to inhibition of bone resorption.

A variety of types of immune cells are present in bone, including macrophages and dendritic cells (DCs), which are terminally differentiated from mature myeloid cells [13]. In our experiments, R848-treated BMMs produced the cytokines IL-6, IL-12, and IFN-γ in culture medium (Figure 2B); a similar phenomenon previously observed with DCs [7]. R848-sensitized BMMs and DCs in bone marrow may suppress the growth of B16F10 cells in bone, as well as by T-cells activated by those cytokines. In addition, we found that intraperitoneal injections of R848 increased the concentrations of IL-6, IL-12, and IFN-γ in serum (Figure 2A), thus it is possible that the growth of transplanted B16F10 cells in bone was held back by R848-induced humoral factors, including IL-6, IL-12, and IFN-γ produced by immune cells in other organs that interact with bone through the circulation. IL-12 and IFN-γ have been shown to have anti-tumor activities, and their level of expression in lymphocytes of cancer patients is low [14]. Additionally, IFN-γ has been adapted for the treatment of kidney cancer. Conversely, IL-6 is regarded as an activator of cancer cells, including the enhancement of invasion activity and cell death resistance [15, 16]. In our study, recombinant IL-6 inhibited the proliferation of B16F10 cells (Figure 3), though use of R848 to up-regulate IL-6 expression may not be appropriate for the treatment of patients with a malignant melanoma. Additional studies are needed in regard to these issues.

An imiquimod 5% cream preparation (Aldara™, 3M, Barcelona, Spain) is used for clinical treatment of such conditions as anogenital HPV-induced warts, anti-tumoral activity in solar keratosis, superficial basal cell carcinoma, and Bowen's disease. Moreover, other reports have indicated that topical treatment with imiquimod cream was an effective therapeutic option for cutaneous metastasis from malignant melanoma [17, 18]. The established safety of these compounds is one reason why imidazoquinoline compounds including R848 are highly anticipated for clinical applications in other diseases, including metastatic malignant melanoma. In addition, another imidazoquinoline compound, 852A, has been shown to inhibit lung colonization by B16F10 cells in mice and was used in a human trial for the treatment of cancer in patients, including those with metastatic malignant melanoma [19, 20]. Even though 43% of those patients had severe adverse events, such as flu-like symptoms, none died during the trial. The present results showed that the recombinant cytokines IL-6, IL-12, and IFN-γ markedly reduced the proliferation of B16F10 cells, while their effects on MMT and 3LL cells were weak (Figure 3). Although R848 might not be effective for a variety of cancer types, its specific effect toward malignant melanoma is noteworthy, along with its reduced side effects on other normal cells. Dummer Reinhard *et al*. reported that 852A treatment prolonged disease stabilization in 19% of patients with stage IV chemotherapy-refractory metastatic malignant melanoma, whereas no clinical response was observed with 852A monotherapy. Our experimental protocol, which started R848 treatment at one day after B16F10 transplantation, showed that the agent markedly inhibited bone invasion by B16F10 cells, indicating that imidazoquinoline compounds may have a substantial inhibitory effect toward metastatic malignant melanoma in an early stage.

Our results also suggest that R848 can inhibit malignant melanoma growth in bone via the activation of the innate immune system. TLR7 is mainly activated by recognizing ssRNAs and imidazoquinoline compounds, and they have different features; ssRNAs strongly induce type-I interferon whereas imidazoquinoline compounds strongly induce pro-inflammatory cytokines such as IL-6 and IL-12 [21, 22]. R848 was originally developed based on imiquimod as a base structure, with the expectation of high anti-viral activity, and was named S-28463 [23]. In fact, R848, which has higher activity compared with imiquimod [24], stimulates immune cells and induces IFN-α, tumor necrosis factor (TNF)-α, IL-6, and IL-12 p40 [24, 25]. In addition, R848 has the advantage that it is less cytotoxic to macrophages than other imidazoquinoline compounds such as imiquimod and gardiquimod [26]. Although additional investigation of important aspects such as administration route, dosage, indications, and reduction of side effects by modifying the chemical structure is required before approval for clinical applications, our findings offer new insights into the possible effectiveness of imidazoquinoline compounds for the suppression of malignant melanoma metastasis into bone tissues.

## MATERIALS AND METHODS

### Reagents

Resiquimod (R848; #SML0196), PD98059 (#P215), SP600125 (#S5567), and LY294002 (#L9908) were purchased from Sigma-Aldrich (St. Louis, MO, US) and BAY11-7082 (#196871) was purchased from Calbiochem (Darmstadt, DE). All drugs were dissolved in dimethyl sulfoxide (DMSO). Quantikine ELISA kits (mouse IL-6, #M6000B; mouse IL-12 p40 non-allele-specific, #MP400; mouse IFN-γ, #MIF00), recombinants (mouse IL-6, #406-ML; mouse IL-12, #419-ML; mouse IFN-γ, #485-MI), and neutralizing antibodies (normal goat IgG control, #AB-108-C; mouse IL-6 antibody, #AF-419-NA; mouse IL-12 antibody, #AF-419-NA; mouse IFN-γ antibody, #AF-485-NA) were purchased from R&D Systems (Minneapolis, MN, US).

### Cells

The mouse malignant melanoma cell line, B16F10, was purchased from the RIKEN Cell Bank (Ibaraki, JP). The mouse spontaneous mammary tumor (MMT) cell line MMT060562 and the Ex-3LL (3LL) mouse Lewis lung carcinoma cell line was purchased from the JCRB Cell Bank (Osaka, JP). RPMI-1640 (Wako: Osaka, JP, #189-02025), RPMI-1640 with MEM Non-essential Amino Acids solution (Wako, #139-15651), and D-MEM (Wako, #044-29765) were used for the B16F10, MMT, and 3LL cells, respectively.

All animal experiments were conducted in accordance with the guidelines of Showa University. C57BL/6J male mice at 8 weeks old were purchased from Sankyo Labo Service Corporation (Tokyo, JP), Inc. Mouse bone marrow cells (BMCs) were collected from the femora and tibiae, from which bone marrow macrophages (BMMs) were formed after culturing in α-MEM (Wako, #135-15175) supplemented with 50 ng/mL of human M-CSF (Leukoprol) (Kyowa Hakko Kogyo: Tokyo, JP) for 3-4 days. All media were supplemented with 10% fetal bovine serum (FBS) and antibiotic-antimycotic solution (Gibco: Waltham, MA, US, #15240-062). Cells were cultured at 37°C in a CO_2_ incubator (5% CO_2_, 95% air).

### Mouse model of melanoma metastasis

Eight-week-old C57BL/6J male mice received intracardiac injections of B16F10 cells (1×10^5^) in 100 μL of PBS under ketamine hydrochloride (100 mg/kg, i.p., KETALAR^®^, DAIICHI SANKYO: Tokyo, JP) and xylazine hydrochloride (10 mg/kg, i.p.; Sigma-Aldrich) anesthesia. Body weights were determined 1, 4, 7, and 10 days later, and mice received i.p. 500 μg injections of the vehicle (DMSO) or R848 (n=7 in each group). On day 13, mice were euthanized and both hindlimbs were obtained. Bone colonization by B16F10 cells was evaluated by counting the numbers of black accumulations in bone epiphyses of the femora and tibiae, with the following used for scoring: 100% = femur and tibia, 50% = femur or tibia, 0% = none.

### Histology

Dissected bones were fixed overnight in 4% paraformaldehyde and decalcified as necessary in 5% EDTA solution before processing, then embedded in paraffin wax. Tibiae sections with a width of 5 μm were stained with hematoxylin and eosin (HE). Images of tibia medial epiphyses were obtained under a Leica DM750 microscope with a Leica MC170HD camera using Leica Application Suite software. The area of invasion by B16F10 cells was evaluated as the area of spreading by those cells per area of the bone marrow cavity. Three samples from each group were chosen for measurements, with three sections of each sample measured.

### Cell proliferation assay

B16F10 cells were seeded into wells of culture plates and cultured until reaching 50% confluence, then R848 or recombinant IL-6, IL-12, or IFN-γ was added to the wells. The next day, an MTS assay was performed using a CellTiter 96^®^ AQueous One Solution Cell Proliferation Assay (Promega: Fitchburg, WI, US, #G3580), according to the manufacturer's instructions.

In another experiment, BMMs were cultured with the vehicle or R848 (100 nM) for one day, then B16F10 cells were cultured in medium supernatant from those BMM cultures for one day and subjected to an MTS assay. In addition, the neutralizing antibodies IL-6 (0.03 μg/mL), IL-12 (0.06 μg/mL), and IFN-γ (1.0 μg/mL) were added to the B16F10 cells cultured in supernatant from the BMM cultures.

### X-ray micro-tomography

Hindlimbs were subjected to three-dimensional μCT with a ScanXmate-L090H (Comscantecno: Kanagawa, JP). Three-dimensional microstructural image data thus obtained were reconstructed using TRI/3D-BON software (Ratoc System Engineering: Tokyo, JP).

### ELISA

Concentrations of IL-6, IL-12 p40, and IFN-γ in serum obtained from 8-week-old C57BL/6J male mice at one day after receiving an intraperitoneal injection of R848 (500 μg) or medium supernatant from BMMs cultured with R848 (100 nM) were measured using a Quantikine ELISA kit, according to the manufacturer's instructions.

### Quantitative RT-PCR

BMMs were treated with the inhibitors PD98059 (12.5 μM), SP600125 (2.5 μM), LY294002 (10 μM), or BAY11-7082 (5 μM) one hour before the culture with R848 (100 nM) for one day.

Total RNA was extracted with TRIzol reagent (Invitrogen), then reverse-transcribed using ReverTra Ace qPCR RT Master Mix (TOYOBO: Osaka, JP). Quantitative real-time PCR (qPCR) was performed using THUNDERBIRD SYBR qPCR Mix (TOYOBO) and the StepOne Real-Time PCR System (Applied Biosystems: Waltham, MA, US). The primer sequences were as follows: *Actb*, 5′-AGATGACCCAGATCATGTTTGAGA-3′ and 5′-CACAGCCTGGATGGCTACGT-3′; *Il6*, 5′-TCGG AGGCTTAATTACACATGTTC-3′ and 5′-TGCCATTG CACAACTCTTTTCT-3′; *Il12b*, 5′-GCACGGCAGCAGA ATAAATATG-3′ and 5′-GGTTTGATGATGTCCCT GATGA-3′; *Ifng*, 5′-TTGGCTTTGCAGCTCTTCCT-3′ and 5′-TGACTGTGCCGTGGCAGTA-3′; and *Ccl2*, 5′-CAGCAGCAGGTGTCCCAAAG-3′ and 5′-TGT CTGGACCCATTCCTTCTTG-3′. Expressions were normalized to that of *Actb*.

### Statistical analysis

Values are expressed as the mean ± standard deviation for each experiment. Student's two-tailed *t*-test was used, with *p* <0.01 considered to indicate significance.

## SUPPLEMENTARY MATERIALS FIGURES



## Abbreviations

TLR: Toll like receptors
BMMs: bone marrow macrophages
DTIC: dimethyl triazeno imidazole carboxamide
PD-1: programmed cell death 1
CTLA-4: cytotoxic T-lymphocyte-associated antigen 4
ssRNA: single-stranded RNAs
pDCs: plasmacytoid dendritic cells
DMSO: dimethyl sulfoxide
MMT: mammary tumor
3LL: Ex-3LL Lewis lung carcinoma
