# Supplementary material for: The inhibition of malignant melanoma cell invasion of bone by the TLR7 agonist R848 is dependent upon pro-inflammatory cytokines produced by bone marrow macrophages
Source: Oncotarget. 2018 Jul 6;9(52):29934–43. doi: 10.18632/oncotarget.25711 (PMC6057452; doi:10.18632/oncotarget.25711)
Supplement: Supplementary file 1 [file oncotarget-09-29934-s001.pdf]

The inhibition of malignant melanoma cell invasion of bone by the TLR7 agonist R848 is dependent upon pro-inflammatory cytokines produced by bone marrow macrophages

SUPPLEMENTARY MATERIALS

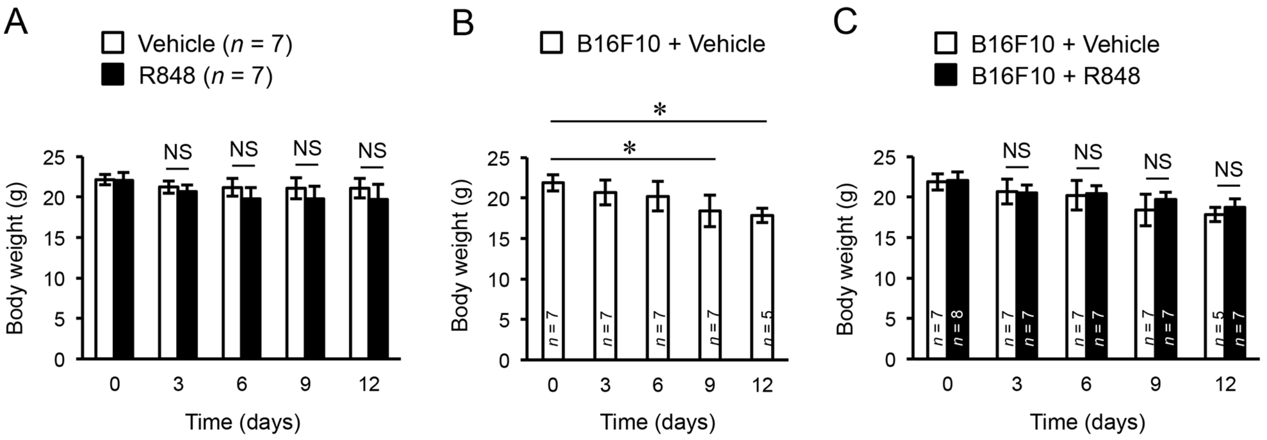

Supplementary Figure 1: R848 administration does not result in altered body weight or rescue loss of body weight caused by transplantation of B16F10 cells. (A) Body weights of mice following intraperitoneal injections with the vehicle (DMSO) or R848 (500 µg) measured every three days. **(B)** Fluctuations in body weights following intracardiac injection of B16F10 cells. **(C)** Body weights of mice injected with B16F10 after intraperitoneal injections with the vehicle or R848 (500 µg) every three days. The data are representative of more than three independent experiments. $^{*}p$ <0.01; NS, not significant.

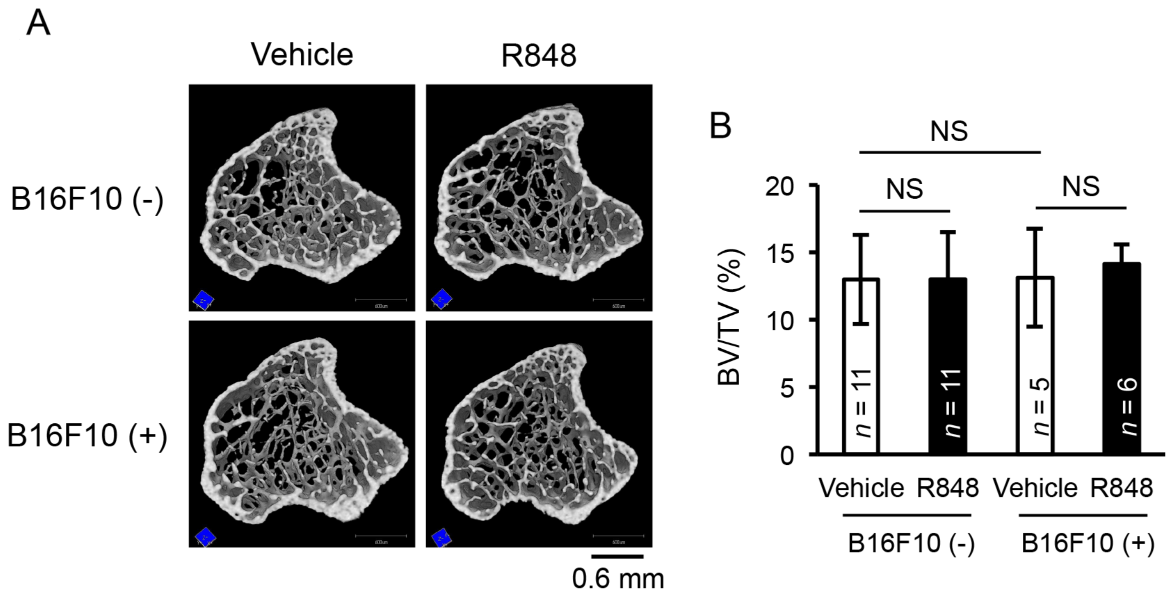

Supplementary Figure 2: R848 does not alter the trabecular bone volume. (A) Representative axial µCT images of tibiae obtained from mice with (+) or without (-) B16F10 cell transplantation, and treatments with the vehicle or R848 (500 µg). **(B)** µCT analysis of bone volume per tissue volume (BV/TV) of tibial trabecular bone. The data are representative of two independent experiments. NS, not significant.
